# Dynamic Epistasis under Varying Environmental Perturbations

**DOI:** 10.1371/journal.pone.0114911

**Published:** 2015-01-27

**Authors:** Brandon Barker, Lin Xu, Zhenglong Gu

**Affiliations:** 1 Center for Advanced Computing, Cornell University, Ithaca, New York, United States of America; 2 Division of Hematology/Oncology, Department of Pediatrics, University of Texas Southwestern Medical Center, Dallas, Texas, United States of America; 3 Division of Nutritional Sciences, Cornell University, Ithaca, New York, United States of America; 4 Tri-Institutional Training Program in Computational Biology and Medicine, New York, New York, United States of America; Universitat Pompeu Fabra, SPAIN

## Abstract

Epistasis describes the phenomenon that mutations at different loci do not have independent effects with regard to certain phenotypes. Understanding the global epistatic landscape is vital for many genetic and evolutionary theories. Current knowledge for epistatic dynamics under multiple conditions is limited by the technological difficulties in experimentally screening epistatic relations among genes. We explored this issue by applying flux balance analysis to simulate epistatic landscapes under various environmental perturbations. Specifically, we looked at gene-gene epistatic interactions, where the mutations were assumed to occur in different genes. We predicted that epistasis tends to become more positive from glucose-abundant to nutrient-limiting conditions, indicating that selection might be less effective in removing deleterious mutations in the latter. We also observed a stable core of epistatic interactions in all tested conditions, as well as many epistatic interactions unique to each condition. Interestingly, genes in the stable epistatic interaction network are directly linked to most other genes whereas genes with condition-specific epistasis form a scale-free network. Furthermore, genes with stable epistasis tend to have similar evolutionary rates, whereas this co-evolving relationship does not hold for genes with condition-specific epistasis. Our findings provide a novel genome-wide picture about epistatic dynamics under environmental perturbations.

## Introduction

Epistasis refers to the phenomenon wherein mutations of two genes can modify each other’s phenotypic outcomes. It can be positive (alleviating), or negative (aggravating), when a combination of deleterious mutations shows a fitness value that is higher, or lower, than expectation, respectively. For example, a mutation that hampers a pathway’s function may allow for other mutations in the same pathway without a fitness consequence, resulting in positive epistasis. Conversely, genes or pathways with redundant functions can give rise to negative epistasis. It is well established that epistasis is important for the evolution of sex [1–3], speciation [4], mutational load [5], ploidy [6], genetic architecture of growth traits [7], genetic drift [8], genomic complexity [9], and drug resistance [10]. As biological systems in nature have to face multiple genetic and environmental perturbations, understanding the global landscape and dynamics of epistasis under these perturbations remains an important issue in the evolutionary field. In an earlier study, we addressed genome-wide epistasis dynamics under various genetic perturbations [11]. In this study, we will investigate the impact of environmental perturbations on global epistasis dynamics.

How epistatic interactions among genes change in different environments has been intensively studied in various model organisms, including *E. coli* [12–14], *S. cerevisiae* [15–17], *C. elegans* [18, 19] and *D. melanogaster* [20–22]. The results of these studies, however, are very controversial. While some studies observed increasing positive epistasis under harsh conditions [13, 17, 20], others have opposite findings [14–16, 18, 19, 21–23]. Even within the same species, different experimental studies might have conflicting conclusions (e.g. [13, 14]). One possible reason for the above controversy could have originated from the fact that most studies only looked at the epistasis dynamics based on a small number of genes, where the properties cannot be generalized to the entire organism.

The main obstacle to exploring global epistatic dynamics under a variety of environments is the difficulty of applying high-throughput experimental platforms. To explore epistasis on a genomic scale, a number of technologies have been developed to systematically map genetic interaction networks, such as synthetic genetic array (SGA) [24, 25], diploid-based synthetic lethality analysis with microarrays (dSLAM) [26, 27], synthetic dosage-suppression and lethality screen [28–30] and epistatic miniarray profiles (EMAP) [31–33]. A key issue for all these experimental studies is that these epistatic networks have been constructed only under normal laboratory conditions. However, cells in nature are constantly bombarded by various external environmental stresses. Epistasis dynamics under these perturbations cannot be predicted based on a single laboratory condition. Few studies have constructed epistatic networks for multiple environments. A recent study that has only constructed epistatic networks for a group of genes with specific functions under one normal and one harsh condition already requires a large amount of effort [34]. Consequently, genome-scale epistasis landscapes under a variety of environmental perturbations remain largely uncharacterized.

Here we explored this issue by using Flux Balance Analysis (FBA) to simulate epistasis dynamics among genes under multiple environmental perturbations. FBA can provide reliable predictions by optimizating a presumed objective function, commonly growth maximization in microbes, subject to the known reactions and constraints of a metabolic network [35–40]. Using this platform, a previous study has investigated synthetic lethal interactions (one type of negative epistasis) under multiple environmental perturbations and showed the plasticity of epistatic interactions in the metabolic networks [41]. Here we examined both positive and negative epistasis using FBA, and were able to show that, on a genome scale, epistatic interactions tend to become more positive in nutrient-limiting conditions relative to abundant-glucose media. In addition, while a large proportion of epistatic interactions can be rewired dynamically under varying environments, there is a set of epistatic interactions that are stable across all tested environments. We also discovered different network and evolutionary properties for genes with stable and dynamic epistatic interactions. Implications of our findings were discussed.

## Methods

Scripts for generating and analyzing the data can be found in the source code repository located at https://github.com/bbarker/COBRAscripts/. Scripts and documentation specific to this paper are located in the subdirectory *MyProjects/EnvironmentalEpistasisFBA*.

### Flux Balance Analysis

Flux Balance Analysis attempts to tackle issues inherent in other methods of metabolic modeling, such as the need to measure a large number of parameters, slow speed of simulation, and dependence on initial conditions [40, 42]. Other than needing a fairly complete understanding of the reactions present in an organism, the only measurements required to perform a genome-scale metabolic simulation are those for determining biomass constitution or a gene expression profile [36, 43]. Strictly speaking, FBA is a particular type of constraint based modeling (CBM). Constraint based modeling frames the stoichiometry that describe the reactions present in an organism as a matrix equation with indeterminates (reaction fluxes) subject to constraints [39, 43]. The optimization problem is described as follows:

$$\begin{array}{cc} \text{maximize} & c^{T}v\\ \text{subject}\;\text{to} & \mathrm{Sv}=\frac{dx}{dt}=0\\ & v_{lb}⪯v⪯v_{ub} \end{array}$$ (1)

**S** is a matrix, in which rows and columns correspond to cellular metabolites and reactions in the reconstructed network respectively. **v** is the reaction flux with upper and lower bounds **v**_*ub*_ and **v**_*lb*_ respectively. Multiplying the stoichiometric matrix **S** by the flux vector v equals the concentration change over time ($\frac{\text{d}x}{\text{d}t}$). At steady state, the flux through each reaction is given by **Sv = 0**. Further details on the underlying methods can be found in the literature [11, 39, 44].

The fluxes of mutations employed in this analysis were restricted to be 50% of the wild-type fluxes found for growth rate maximization by geometric FBA [44]. To find new conditions with a specified carbon source or other limiting nutrient that achieves 20% of the high-glucose growth rate, we can solve a linear program for the minimization of the limiting nutrient uptake while requiring the growth rate to be equal to 20% of the abundant-glucose growth-rate. For maximum growth rate conditions (S1 Table, S2 and S4 Figs.), we allowed unrestricted uptake of the limiting nutrient to obtain the maximum growth in that condition, up to the point where it would reach the high-glucose growth rate. Mutations affecting protein complexes and pleiotropic genes are handled by uniform restriction across enzymes as described before [11].

### Definition of epistasis

In each gene mutant pair, the epistasis value is calculated based on the equation: *ϵ* = *W_xy_ − W_x_W_y_*, in which *W_xy_* is the fitness of an organism with two mutations in genes X and Y, whereas *W_x_* or *W_y_* refers to the fitness of the organism with mutation only at gene X or Y respectively. Each fitness listed previously is calculated relative to the wild-type fitness. Absolute fitness values are determined by the value of the biomass maximization objective present in the model. Finally, a confidence threshold (|*ϵ*| ≥ 0.01) was applied to generate epistatic interactions [11, 25, 44]. We also conducted analyses based on a different threshold for epistasis and the general conclusions still hold in our analysis (S1 and S3 Figs.).

### Evolutionary rates and network parameters

Evolutionary rates of *S. cerevisiae* genes were downloaded from supplementary materials of [45], in which orthologs were defined by four complete genomes of *Saccharomyces* species (*Saccharomyces cerevisiae*, *Saccharomyces paradoxus*, *Saccharomyces mikatae* and *Saccharomyces bayanus*) and evolutionary rates at synonymous and nonsynonymous sites were calculated based on a four-way yeast species alignment for *S. cerevisiae* genes by PAML. For the distributions in 5A, we randomly sampled gene pairs with the same number of gene pairs as in three epistasis networks (epistasis in all 16 conditions, extremely stable epistasis, and extremely dynamic epistasis, respectively), and calculated the average evolutionary rate differences between random gene pairs in each of these three sample sets. The simulations were repeated 10,000 times for each of the three groups, which are color coded to correspond to the epistasis networks of the same size.

Network parameters such as the shortest path length, clustering coefficient and closeness were calculated using the computer software Pajek, downloaded from: http://vlado.fmf.uni-lj.si/pub/networks/pajek. The shortest path length between two genes in a network reflects the overall network interconnectedness; the smaller the average shortest path length is, the higher chance that genes in this network could interact with the other genes. The clustering coefficient of a network is a measurement of the degree to which nodes in a network tend to cluster together; the larger the average clustering coefficient is, the more closely the genes are connected, forming modules. The closeness of a network measures the centrality of nodes within a network; nodes that occur on shortest paths with other nodes have higher closeness than those that do not [46].

## Results

### FBA modeling and simulated growth conditions

We applied the yeast *S. cerevisiae* metabolic reconstruction iMM904 [43] to examine the dynamics of epistasis under various environmental perturbations. The reconstruction has 904 metabolic genes that are associated with 1,412 metabolic reactions. We conducted FBA simulations under an abundant-glucose condition and 16 nutrient-limiting conditions. In 15 of these conditions, the carbon source (abundant glucose) was replaced by one of the following: acetaldehyde, acetate, adenosine 3',5'-bisphosphate, adenosyl methionine, adenosine, alanine, allantoin, arginine, ethanol, glutamate, glutamine, glycerol, low glucose, trehalose, and xanthosine, respectively. These conditions represent a wide variety of nutrient and energy sources: nucleosides, amino acids, sugars, alcohols, etc. Additionally, we looked at abundant glucose under limited phosphorus availability.

To ensure that all environments have the same growth rates in the following analyses, we restricted the carbon source or phosphorous uptake levels for each of the 16 environmental perturbations such that only 20% of the high-glucose growth rate was attained. This was chosen because it has been shown that metabolism is directly linked to growth and similar growth rates often induce similar metabolic pathways [47]. It is therefore important to use a fixed growth rate among different conditions to control for the relationship between growth rates and the overall metabolic activity so as not to induce a growth-rate specific effect. The 20% high-glucose level was chosen because some media types do not support high growth rates, regardless of the abundance of the nutrient source. Specifically, the acetate condition had the lowest wild-type growth rate (38.5% of the wild-type glucose growth rate) when unrestricted carbon uptake was permitted. In order to allow flexibility with adding more conditions in future studies while simultaneously not allowing extremely low growth rates that may be possible to model but are unlikely to persist in natural environments, we chose a growth rate of approximately half of this minimal wild-type growth rate as the the wild-type growth rate for all conditions.

In order to estimate epistasis between genes, we created a mutation for each gene in each condition that restricted the flux to be 50% of the wild-type flux found by geometric FBA for all reactions associated with the mutant gene [11]. Epistatic relations between any two genes were calculated under each condition. We also tested our core findings allowing maximum growth in each condition (S1 Table) and the general trends in our results remained similar, as described in the following.

### More positive differential epistases from rich media to nutrient-limiting conditions

**Figure 1:**
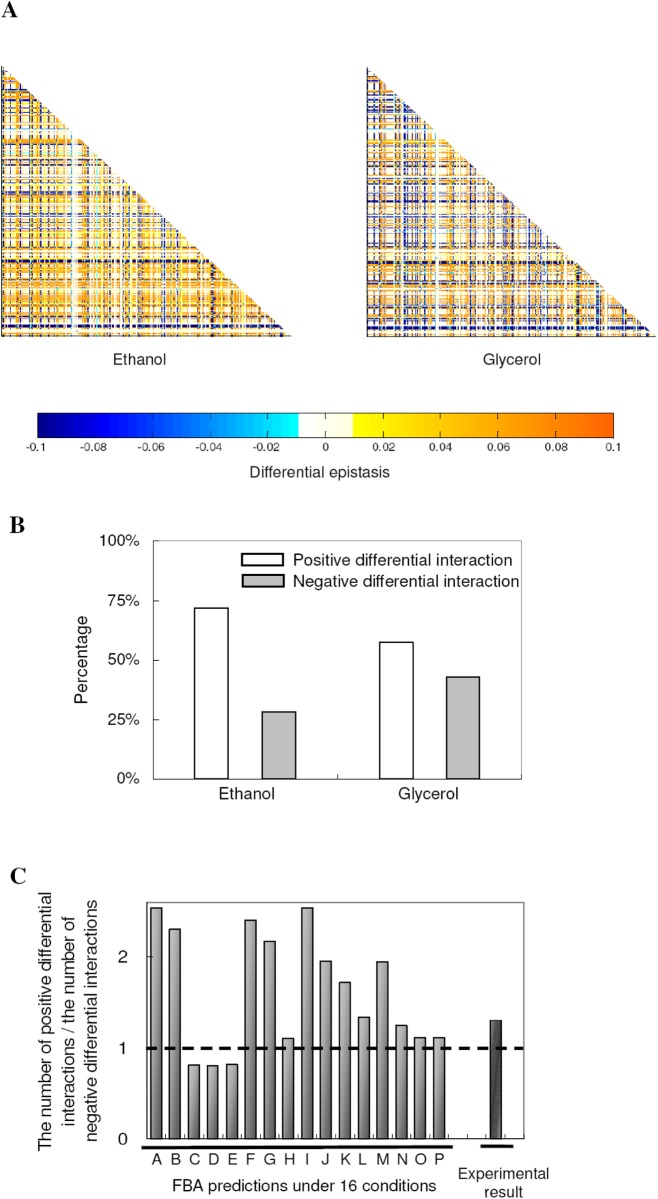
More positive differential epistases under environmental perturbations. (**A**) Heat maps describe the global dynamics of differential epistasis from abundant-glucose medium to ethanol (left panel) and glycerol (right panel) conditions. Only gene pairs with |d*ϵ*| ≥ 0.01 in either condition are included in the figure. Different colors represent differential epistasis values as indicated by the color bar at the bottom. The differential epistasis values are assigned to be 0.1 (or -0.1) in the heat-maps when it is greater than 0.1 (or less than -0.1). It is noteworthy to point out that the epistasis patterns are indeed very different between the two conditions (Fig. 2A). (**B**) Percentage of positive and negative differential epistases under ethanol and glycerol conditions. (**C**) Ratio of positive to negative differential epistases in each simulated condition. The result from a high-throughput experiment is also shown. The letters A-P represent acetaldehyde, acetate, adenosine 3',5'-bisphosphate, adenosyl methionine, adenosine, alanine, allantoin, arginine, ethanol, glutamate, glutamine, glycerol, low glucose, phosphate, trehalose, and xanthosine, respectively.

To directly address how the sign and magnitude of epistases change under nutrient-limiting conditions, we calculated differential epistasis (d*ϵ*), which is defined as the epistatic change from abundant-glucose media to the nutrient-limiting condition for each gene pair in each growth condition. A gene pair with positive (or negative) differential interaction under an environmental perturbation is defined as the gene pair having increasing (or decreasing) epistasis values from the abundant-glucose media to that condition. Fig. 1A depicts the distribution of differential epistases in two growth conditions (ethanol and glycerol) as an example. Only genes with |d*ϵ*| ≥ 0.01 in at least one of the two conditions are included in this figure. As quantified in S2 Table, there are 6.1% and 5.5% of all gene pairs with |d*ϵ*| ≥ 0.01 from abundant-glucose media to ethanol and glycerol growth conditions, respectively. Among them, a large number of gene pairs even change their sign of epistasis (S2 Table). Simulations in other conditions show similar effects (S2 Table), indicating that epistatic relationships among genes can be very dynamic between abundant-glucose media and nutrient-limiting conditions.

We further investigated the sign of differential epistasis from abundant-glucose to nutrient-limiting conditions. As shown in Fig. 1A, we observed more yellow dots (positive differential epistasis) than blue dots (negative differential epistasis) in both panels. Indeed, as quantified in Fig. 1B, 72% and 57% of differential epistases are positive in ethanol and glycerol conditions, respectively. We further explored all 16 nutrient-limiting conditions and the results are shown in Fig. 1C. In most of our simulated conditions (13/16), there are significantly more positive differential epistases than negative differential epistases (Binomial test, P < 10^−5^ for each of the 13 conditions), indicating that epistasis tends to become more positive in nutrient-limiting conditions. This conclusion does not depend on the criteria we used to define differential epistasis (S1 Fig.).

A recent high-throughput experiment measured epistatic relations between roughly 80,000 gene pairs with and without perturbation by a DNA-damaging agent (methyl methanesulfonate, MMS). The study represents the most comprehensive experimental study so far to explore epistatic dynamics from a rich medium to a harsh condition [34]. Interestingly, the authors also found more positive differential epistases than negative differential epistases, which is consistent with our general observation (Fig. 1C). We further allowed maximum growth in each condition and the general trends in our results remained similar (S2 Fig.).

We found that differential epistasis had functional importance after performing both Gene Ontology (GO) and Kyoto Encyclopedia of Genes and Genomes (KEGG) enrichment analyses to compare genes with positive and negative differential epistases through the glucose-abundant to ethanol transition. We chose the ethanol condition as an example because it is one of the most widely used conditions for the baker’s yeast. We observed that 38 GO terms and 8 KEGG pathways are enriched for positive differential epistasis, while 18 GO terms and 1 KEGG pathways are enriched for negative differential epistasis (S3 Table). More importantly, we found positive and negative differential epistases uniquely contribute to different aspects of ethanol and energy metabolism. For example, positive differential epistasis is enriched in monohydric alcohol metabolic processes, oxidoreductase activity acting on aldehyde group donors, the TCA cycle, and pyruvate metabolism, while negative differential epistasis is enriched in ethanol metabolic processes and various amino acid terms and pathways, indicating the functional importance of differential epistasis (S3 Table). This is consistent with experimental results that show differential epistatic interactions, rather than static epistatic interactions, are functionally related to the response of interest [34].

Several system properties were found to correlate with the ratio of the number of positive to negative differential epistases (S4 Table). A strong correlation exists between the number of essential genes in a given condition and the ratio of positive to negative differential epistasis on transition from high glucose to that condition (*ρ* = 0.8392, P = 4.8107 × 10^−5^), which was a better predictor than the number of non-zero fluxes in the wild-type vector for that environment (*ρ* = 0.5115, P = 0.0428). Another strong predictor for positive differential epistasis was the mean relative fitness of single mutants in the new environment (*ρ* = −0.8029, P = 2.7427 × 10^−4^); this anticorrelation suggests that a propensity for a lower single mutant fitness can cause a shift towards positive epistasis.

### Dynamic epistasis between nutrient-limiting conditions

**Figure 2:**
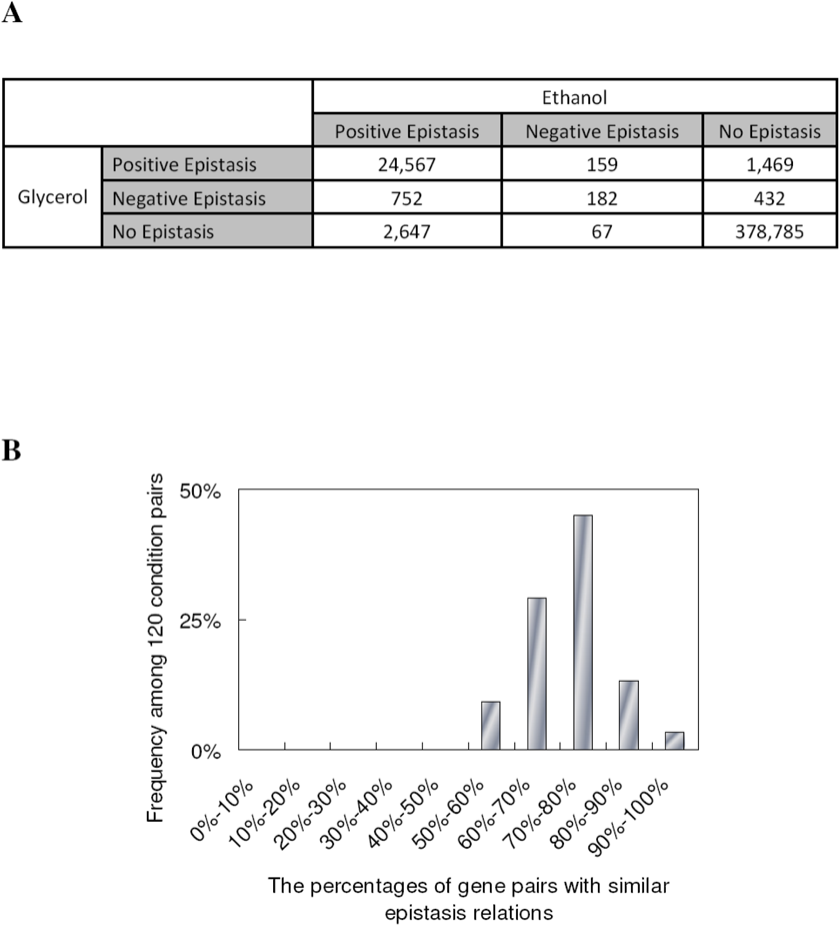
Epistasis dynamics between environmental perturbations. (**A**) Number of gene pairs with various epistatic relationships between ethanol and glycerol growth conditions. (**B**) The distribution for the percentages of gene pairs with similar epistasis relation between any 2 of 16 conditions. The frequency is derived from the 120 pairs of environments simulated in this study.

Fig. 1 explored the epistasis dynamics from abundant-glucose media to nutrient-limiting conditions. As biological systems in nature constantly face perturbations to the environment, it is interesting to investigate the epistasis dynamics among nutrient-limiting conditions. To achieve this aim, we first explored the epistatic relationship between the same gene pairs in ethanol and glycerol environments. Fig. 2A lists the number of gene pairs that have various epistatic relationships. It is noteworthy to point out that, consistent with previously published results, there are significantly more positive epistases than negative epistases between genes in either condition [44].

If two genes have the same sign of epistasis and |*ϵ*| ≥ 0.01 in both conditions, they are defined as having a similar epistatic relationship in these two conditions. To quantify epistatic dynamics between ethanol and glycerol growth conditions, we defined the percentage of gene pairs with similar epistatic relations to be the number of gene pairs with similar epistasis relations shared in these two conditions (overlap) divided by the number of gene pairs with epistasis in either condition (union). Our results show that 79% of gene pairs have similar epistasis relations between these two conditions. Fig. 2B shows the distribution for the percentages of gene pairs with similar epistasis relations between any 2 of 16 conditions, demonstrating a variable degree of epistatic similarity between any two conditions. This conclusion still holds when we used different criteria to define epistatic relationships between genes (S3 Fig.).

**Figure 3:**
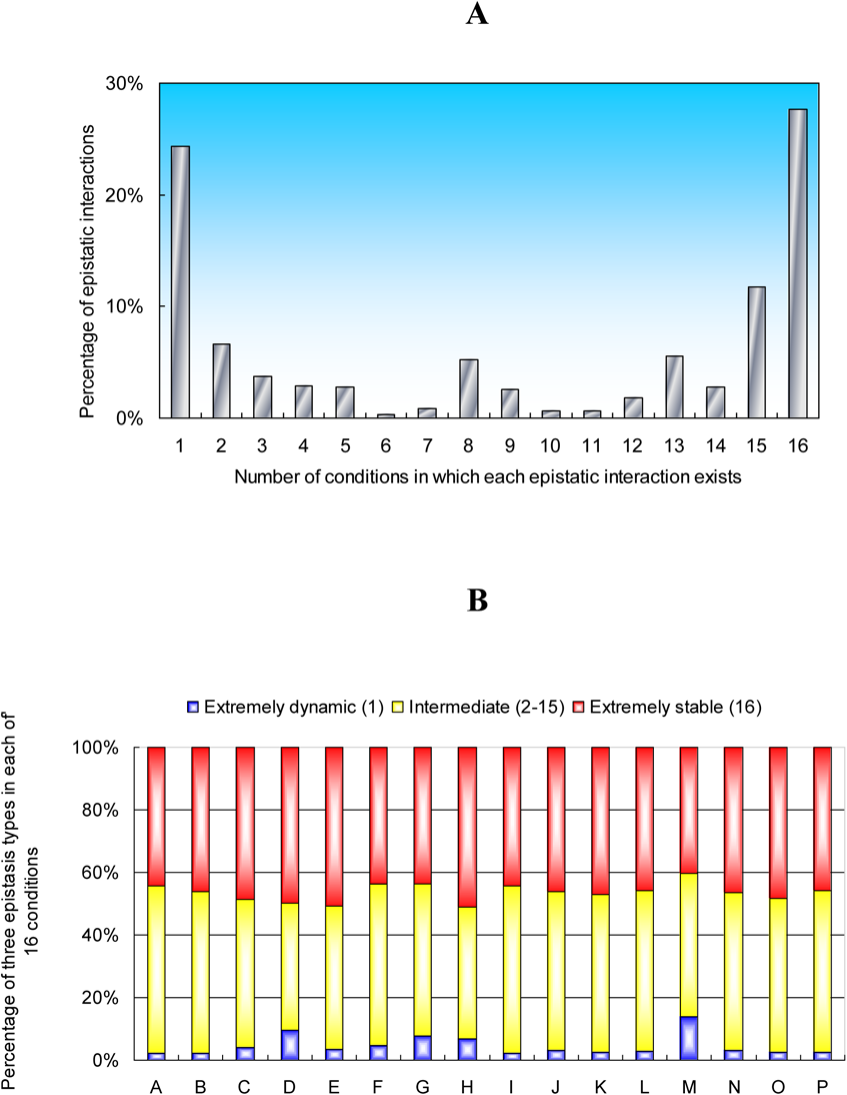
The global distribution of epistatic relations under simulated conditions. (**A**) Distribution for the number of conditions in which each epistatic interaction exists. Note that about 28% of epistatic relations are extremely stable (the very right bar) and about 24% are extremely dynamic (the very left bar). (**B**) Fraction of three types of epistatic relations in each of the 16 environmental perturbations, as indicated by the color bar to the right. The numbers in the brackets represent the number of conditions in which each epistatic interaction exists, as indicated in (**A**). The letters A-P represent the simulated conditions as indicated in Fig. 1.

To understand the global distribution of all epistatic relations, we considered 16 conditions together and calculated the fraction of epistatic interactions existing in 1, 2, 3, …, 15, and 16 conditions, respectively. As shown in Fig. 3A, we found that there is a U frequency distribution for the number of growth conditions in which a specific epistatic interaction is observed. This means that approximately 52% of these interactions are either condition-specific (24%; termed dynamic) or predicted to exist in all conditions (28%; termed stable), and about 48% is intermediate (exists in multiple but not all 16 conditions). An analogous result was obtained previously, but only for synthetic lethal interactions [41]. We also changed the growth assumption and allowed maximum growth in each condition and reanalyzed the global distribution of all epistatic relations. The U frequency distribution for the number of growth conditions in which a specific epistatic interaction is observed remained similar (S4 Fig.). Based on the result in Fig. 3A, we further calculated the ratio of these three types of epistatic relations in each of the 16 environmental perturbations. As shown in Fig. 3B, we found that in each environment, about 40–60% of epistatic interactions are stable and that each environment also has many private epistases among genes.

### Different network properties for stable and dynamic epistasis

**Figure 4:**
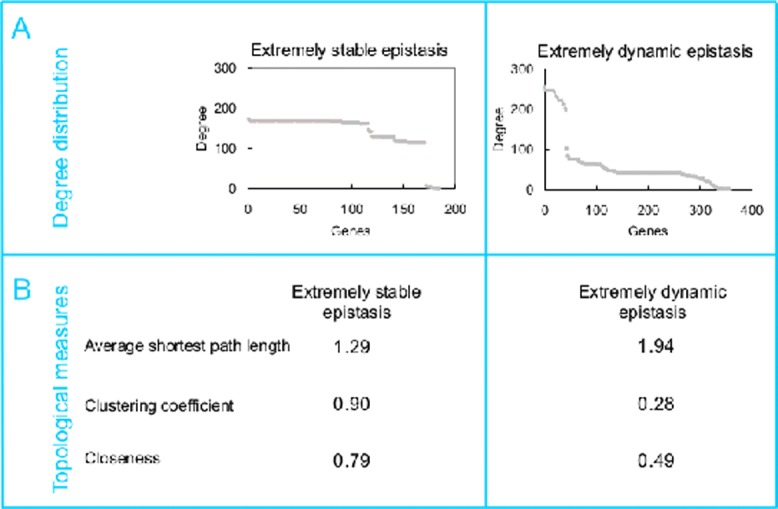
Network properties for the extremely stable and extremely dynamic epistatic interactions. (**A**) Degree distribution for genes in two epistatic interaction networks. The networks have nodes that correspond to genes and edges that correspond to epistatic interactions. (**B**) Three network parameters (the definition of which are shown in Methods) for two epistatic interaction networks.

Analysis on network properties can reveal various organization principles (e.g. frequency of occurrence, centrality) for epistasis networks [24, 25] and therefore provide valuable information to further distinguish stable and dynamic epistasis. To achieve this aim, we compared networks formed by extremely stable and extremely dynamic epistasis among genes and asked whether they have distinct network properties. The degree distributions for both types of epistasis are shown in Fig. 4A. Interestingly, extremely stable epistatic interactions form an exponential network architecture, which is homogeneous, meaning that most nodes have a very similar number of links ( Fig. 4A, left panel). In contrast, the extremely dynamic epistatic interactions give rise to a scale-free network topology, which is heterogeneous, meaning that the majority of nodes have few links but a small number of hubs have a large number of links (Fig. 4A, right panel).

In addition, we calculated three network parameters to compare these two types of epistatic interactions. We found that the network formed by extremely stable epistases has a smaller shortest path length, a larger clustering coefficient and larger closeness than the network formed by extremely dynamic epistases (Fig. 4B). These results are consistent with the scenario that genes with extremely stable epistasis are directly linked to most other genes and form an exponential network topology, while genes with extremely dynamic epistasis form a scale-free network. Our results also show that the network induced by intermediate epistases have intermediate values of these parameters compared to that of extremely stable and extremely dynamic epistasis networks (S5 and S6 Tables).

### Co-evolution of genes with epistatic interaction

**Figure 5:**
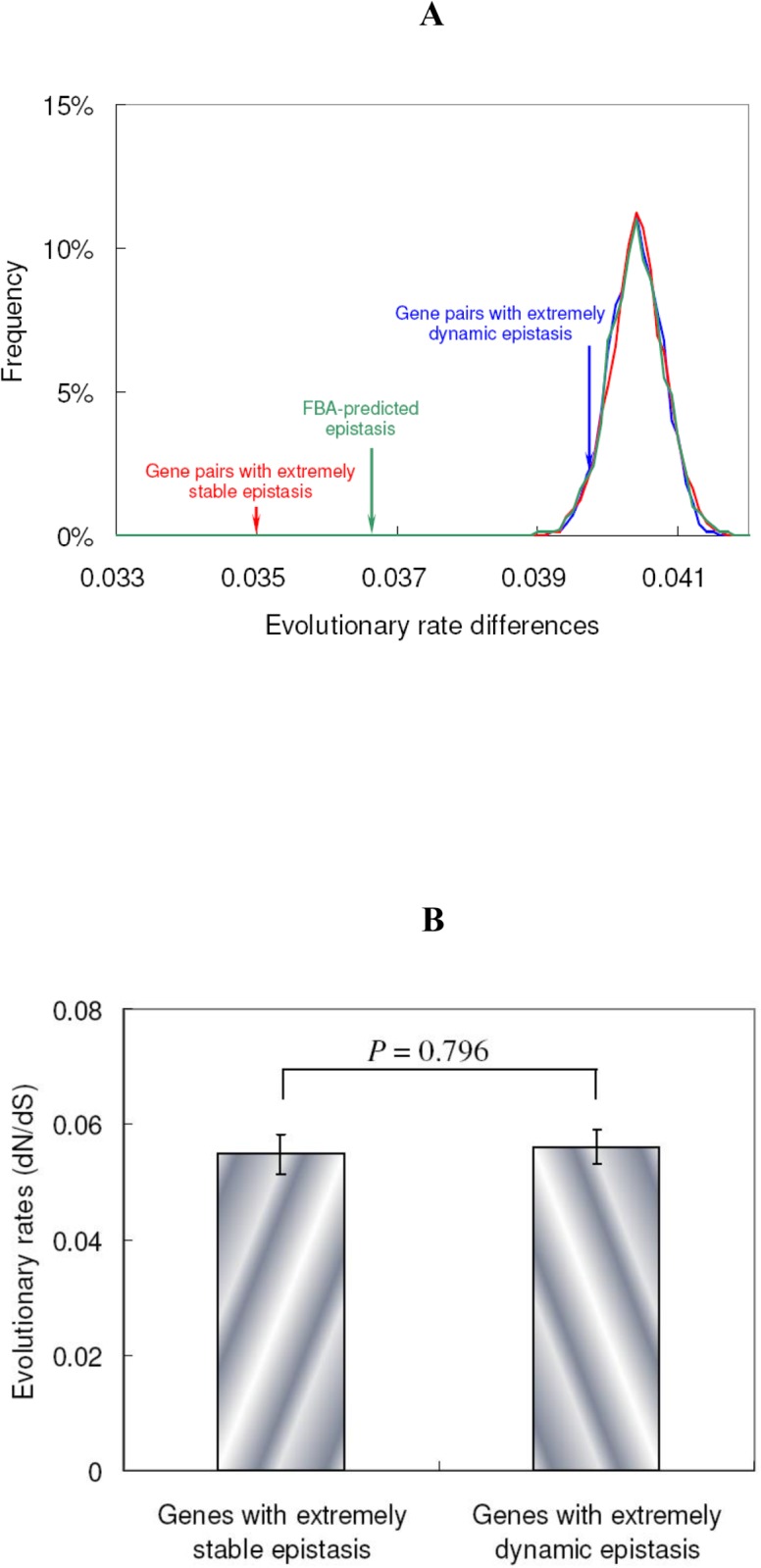
Co-evolution between genes with epistasis. (**A**) Average evolutionary rate differences between gene pairs with FBA-predicted epistasis (green), extremely dynamic epistasis (blue) and extremely stable epistasis (red) are highlighted by three arrows, respectively. The random simulations with the same number of gene pairs as each of the three groups were repeated 10,000 times and the frequency distributions are shown (marked by the same colors as the corresponding arrows, respectively). (**B**) The evolutionary rates for genes that are involved in extremely stable and extremely dynamic epistasis, respectively. The error bars represent standard errors.

Gene pairs with epistasis identified in real experiments usually show similar evolutionary rates. To investigate whether two genes with predicted epistasis also tend to co-evolve, we calculated the evolutionary rate differences between two genes with epistasis from FBA modeling (Fig. 5A). Evolutionary rates (*d*_N_/*d*_S_) based on orthologous gene sets from four yeast species of the genus Saccharomyces were downloaded from a commonly used reference dataset [45]. Simulations based on the same number of gene pairs with FBA-predicted epistasis were conducted to estimate the evolutionary rate differences for any two randomly selected genes. As shown in Fig. 5A, the gene pairs with FBA-predicted epistatic interactions tend to have more similar evolutionary rates than random expectation (P < 10^−4^).

In Fig. 4 we observed unique network properties for extremely stable and extremely dynamic epistatic interactions. We further investigate the co-evolution between genes with these two types of epistatic relationships. As shown in Fig. 5A, genes with extremely stable epistasis tend to co-evolve (P < 10^−4^), while the difference between genes with extremely dynamic epistasis and random expectation becomes much smaller (P = 0.06). The evolutionary rate difference between gene pairs with extremely stable and extremely dynamic epistasis is also significant (*t*-test, P = 8 × 10^−6^). This difference is not caused by genes that are involved in extremely stable or extremely dynamic epistasis, because these two groups of genes do not have significantly different evolutionary rates (*t*-test, P = 0.796, Fig. 5B).

## Discussion

### Natural selection in nutrient-limiting conditions

Whether a genetic mutation has a fitness consequence depends on other sites, a phenomenon called epistasis (see [48] for a recent review on molecular mechanisms). Positive epistasis alleviates the total harm when multiple deleterious mutations combine together and thus reduces the effectiveness of natural selection in removing these deleterious mutations, whereas negative epistasis plays the opposite role by increasing the efficiency of purging deleterious mutations by natural selection. Results from this study present an initial glimpse over environment-induced epistasis dynamics at the genome scale. Using differential epistasis from abundant-glucose to nutrient-limiting conditions, our results show that epistasis between specific genes can become more positive or more negative in nutrient-limiting conditions, which is consistent with previous findings in small scale studies [12–23]. However, we showed that, at the genome scale, epistasis is more positive in nutrient-limiting conditions. Interestingly, our simulation results are consistent with a recent genome-wide study between laboratory and harsh growth conditions [34]. How epistasis affects selection in harsh conditions has been controversial [49]. Our results provide the genome-wide evidence arguing that selection might be less effective in removing deleterious mutations in harsh conditions, which could be one of the underlying reasons for a recent observation that stimulation of a stress response can reduce mutation penetrance in *Caenorhabditis elegans* [50].

### Network properties and evolutionary patterns for stable and dynamic epistasis

Our results indicate that epistasis could be extremely stable or dynamic among various environmental perturbations, which is consistent with a previous FBA study investigating synthetic lethal relations among non-essential genes [41]. The inclusion of essential genes in our study allows for investigation on many important metabolic pathways that were not previously analyzed. Nevertheless, the distribution of epistasis among multiple environments (Fig. 3A) remains largely unchanged from the previous study [41] even when essential genes are included.

We also found that stable and dynamic epistatic relationships show totally different network properties and evolutionary patterns, which might provide new biological and evolutionary insights. The gene pairs with stable epistases tend to co-evolve with each other. In addition, from the biological pathway perspective, the smaller shortest path length and larger closeness values in the stable epistasis network both imply that genes with stable epistases tend to be functionally associated with a large number of neighbors to form a condensed functional network, different from genes in the dynamic epistasis network that are loosely connected. Furthermore, the large clustering coefficient in the stable epistasis network also supports the idea that genes with stable epistasis interactions form a network core in the whole epistasis network. Combined with observations in Fig. 5, this core module of epistasis in the metabolic network might represent stable functional associations between genes that are essential for important biological functions and evolutionarily conserved even under different environmental perturbations. The lack of co-evolutionary pattern and scale-free network properties for the dynamic epistasis network, however, might represent unstable functional associations between genes, which may only be responsible for unique functions under specific conditions.

### Implications and significance for exploring stable and dynamic epistasis

Our prediction about stable and dynamic epistasis could have important functional applications. A recent study showed that the synthetic lethal (negative epistasis) relationship between fumarate hydratase and haem oxygenase can be employed successfully to identify an in vitro drug target in hereditary leiomyomatosis and renal-cell cancer (HLRCC) cells [51]. Exploring both dynamic and stable epistasis could be useful in this context; stable epistatic interactions may be important for drug target detection in cancer or other pathogens, whereas it may sometimes be necessary to exploit dynamic epistatic relationships, possibly induced by treatment with an external perturbation.

Furthermore, rational evolutionary design techniques such as OptKnock [52] and OptGene [53] attempt to find which knockouts will enable a reaction of interest to be coupled with growth (i.e. have positive epistasis with growth-associated genes in a specific environment). However, these techniques do not take into account epistasis dynamics across different environments. In this study, we have found that epistatic relations can be highly dynamic under various environmental perturbations, which raises the possibility to improve these techniques by considering epistasis dynamics in future studies. Research on using compensatory perturbations to reach desired network states is ongoing [54].

### Caveats and future directions

Though we show several novel insights into how varying environments can influence epistasis, several caveats should be addressed. First, the FBA modeling used in this study, which was proven to have great predictive power and has been successfully employed in addressing numerous research problems [35, 36, 39], only includes metabolic genes. Second, even though FBA offers the most comprehensive simulation method for studying epistasis, there are many improvements that can be made in order to capture the empirically observed set of epistatic interactions [55]. For example, integrating transcriptional regulation and physical interactions into this framework could improve the current methods in predicting epistasis and other evolutionary processes [56]. Related to this point, FBA as used herein only considers the steady state and does not take into account any dynamics or initial conditions, and would necessarily miss any epistatic interactions that are due to dynamics in the system, such as changing concentrations; dynamic FBA (which is part of rFBA) might be a solution, but would likely require about a minimum of two orders of magnitude increase in computation time [55, 57]. Recent work on new objective functions targeting metabolite turnover rather than flux per se has also proven successful in recovering many epistases that were previously not found with FBA [58].

Third, in order to understand the impact of environmental perturbations on epistasis, we used a reductive approach and only considered one mutation type per gene to simulate the global epistatic landscape in 16 environments. There are countless environments in nature. Furthermore, different mutations in the same gene and the interactions between genes and environment can likely have an even more complex impact on the epistasis dynamics. While it would be ideal to simulate a larger variety of environments for multiple mutations of the same gene, the computational cost is a limiting factor. Our previous study showed that different mutants of the same gene could have very dynamic epistatic interaction partners in a single environment [11]. In this study, we chose to use one mutation per gene as we are focusing on addressing how different environments could affect gene epistasis dynamics. Nevertheless, in order to see how sensitive our results were, we performed the analysis for our core results by simulating 16 environments using different growth assumption, where the organisms are allowed to have unrestricted uptake of the limiting nutrient to obtain the maximum growth in that condition. We found the major trends in our results are largely unchanged (S2 and S4 Figs.; S1 Table).

Keeping these issues in mind, our analysis uncovered several prominent features of epistatic interactions under a variety of environmental perturbations, and call on future effort to confirm these simulation results using high-throughput experimental platforms. More importantly, the enrichment of stable and dynamic epistasis provides a new perspective to understand how biological systems may rewire epistasis in nature.

## Supporting Information

S1 Fig More positive differential epistases under environmental perturbations for different thresholds of differential epistasis (|d*ϵ*| ≥ 0.01, A) and (|d*ϵ*| ≥ 0.05, B). Ratio of positive to negative differential epistases in each simulated condition are shown. The letters A-P represent acetaldehyde, acetate, adenosine 3',5'-bisphosphate, adenosyl methionine, adenosine, alanine, allantoin, arginine, ethanol, glutamate, glutamine, glycerol, low glucose, phosphate, trehalose, and xanthosine, respectively.
(TIF)

S2 Fig Analogous to Fig. 1B–C, but using a maximum growth rate for each condition, where the maximum is constrained to be no higher than the high-glucose growth rate. (**A**) Percentage of positive and negative differential epistases under ethanol and glycerol conditions. (**B**) Ratio of positive to negative differential epistases in each simulated condition. The result from a high-throughput experiment is also shown. The letters A-P represent acetaldehyde, acetate, adenosine 3',5'-bisphosphate, adenosyl methionine, adenosine, alanine, allantoin, arginine, ethanol, glutamate, glutamine, glycerol, low glucose, phosphate, trehalose, and xanthosine, respectively. Note that in (B), low glucose has the same growth rate as high-glucose, but has different epistatic interactions since we still use the high-oxygen uptake level associated with the low glucose condition.
(TIF)

S3 Fig Epistasis dynamics between environmental perturbations under different epistasis definition. (**A**) Number of gene pairs with various epistatic relationships between ethanol and glycerol growth conditions under a lower (|*ϵ*| ≥ 0.01) and a higher (|*ϵ*| ≥ 0.05) epistasis threshold. (**B**) The distribution for the percentages of gene pairs with similar epistasis relations between any 2 of 16 conditions under a lower (|*ϵ*| ≥ 0.01) and a higher (|*ϵ*| ≥ 0.05) epistasis threshold.
(TIF)

S4 Fig Analogous to Fig. 3A, but using a maximum growth rate for each condition, where the maximum is constrained to be no higher than the high-glucose growth rate. Distribution for the number of conditions in which each epistatic interaction exists. Note that about 26% of epistatic relations are extremely stable (the very right bar) and about 19% are extremely dynamic (the very left bar).
(TIF)

S1 Table Wild-type growth rates used in the maximal growth rate simulations used for S2 and S4 Figs. (XLSX)

S2 Table Condition-specific epistases and sign-epistases prevalence in the iMM904 yeast model. (XLSX)

S3 Table GO term enrichment analysis results for differential epistasis in transition to ethanol. (XLSX)

S4 Table Properties of simulated systems that correlate with the ratio of positive to negative differential epistases. (XLSX)

S5 Table List of epistatic interactions for the extremely stable, dynamic, and intermediate epistasis networks. (XLSX)

S6 Table Table of network parameters for stable, dynamic, and intermediate epistasis. (XLSX)
