# Supplementary figures and images for: Dynamic Epistasis under Varying Environmental Perturbations
Source: PLoS One. 2015 Jan 27;10(1):e0114911. doi: 10.1371/journal.pone.0114911 (PMC4308068; doi:10.1371/journal.pone.0114911)

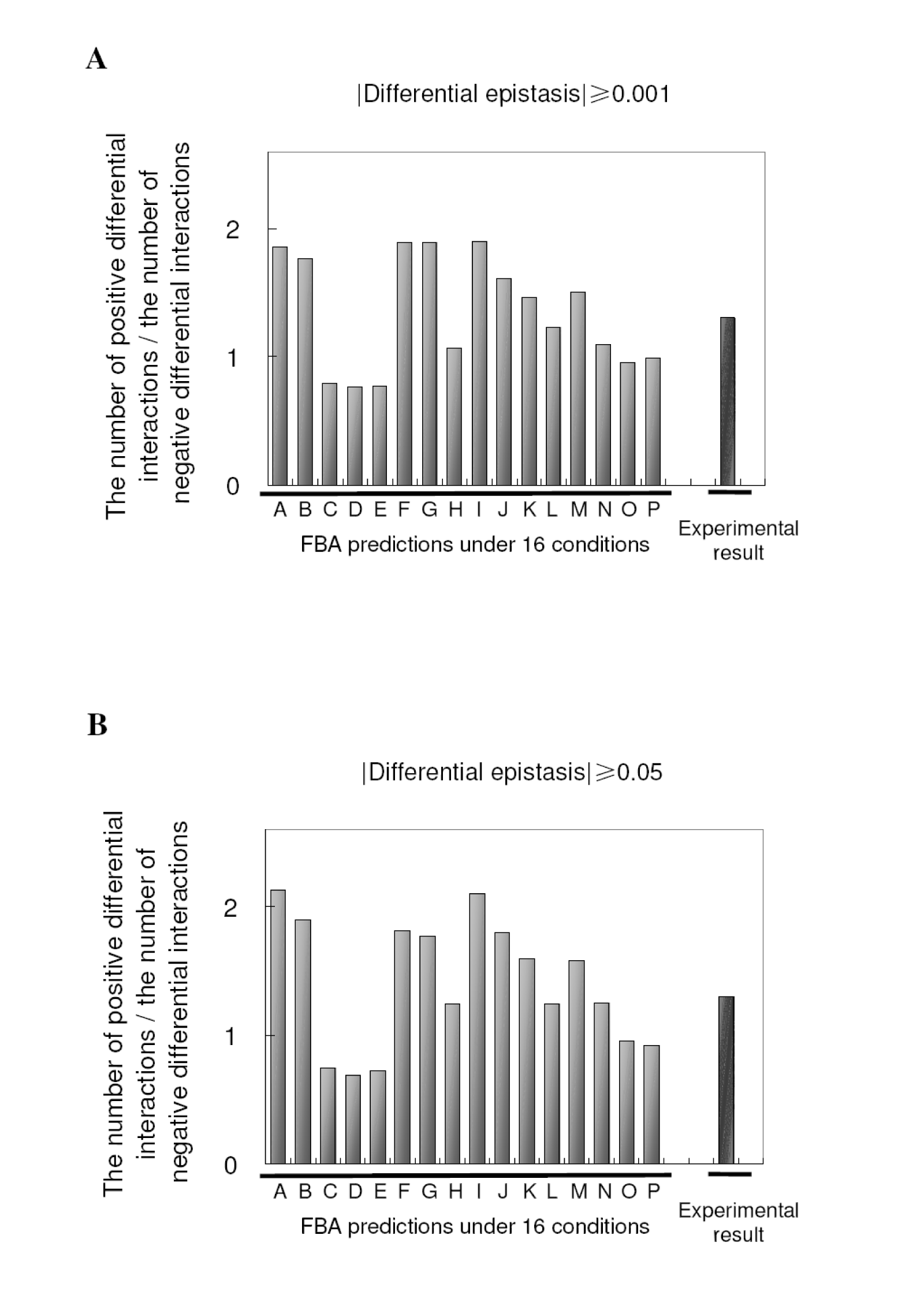

Supplement: S1 Fig — Ratio of positive to negative differential epistases in each simulated condition are shown. The letters A-P represent acetaldehyde, acetate, adenosine 3',5'-bisphosphate, adenosyl methionine, adenosine, alanine, allantoin, arginine, ethanol, glutamate, glutamine, glycerol, low glucose, phosphate, trehalose, and xanthosine, respectively. (TIF) [file pone.0114911.s001.tif]

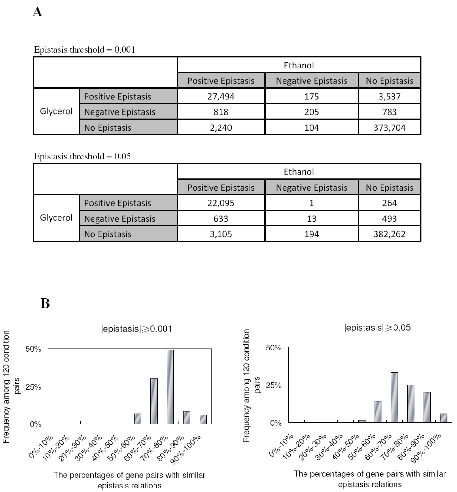

Supplement: S3 Fig — (A) Number of gene pairs with various epistatic relationships between ethanol and glycerol growth conditions under a lower (|ϵ| ≥ 0.01) and a higher (|ϵ| ≥ 0.05) epistasis threshold. (B) The distribution for the percentages of gene pairs with similar epistasis relations between any 2 of 16 conditions under a lower (|ϵ| ≥ 0.01) and a higher (|ϵ| ≥ 0.05) epistasis threshold. (TIF) [file pone.0114911.s003.tif]

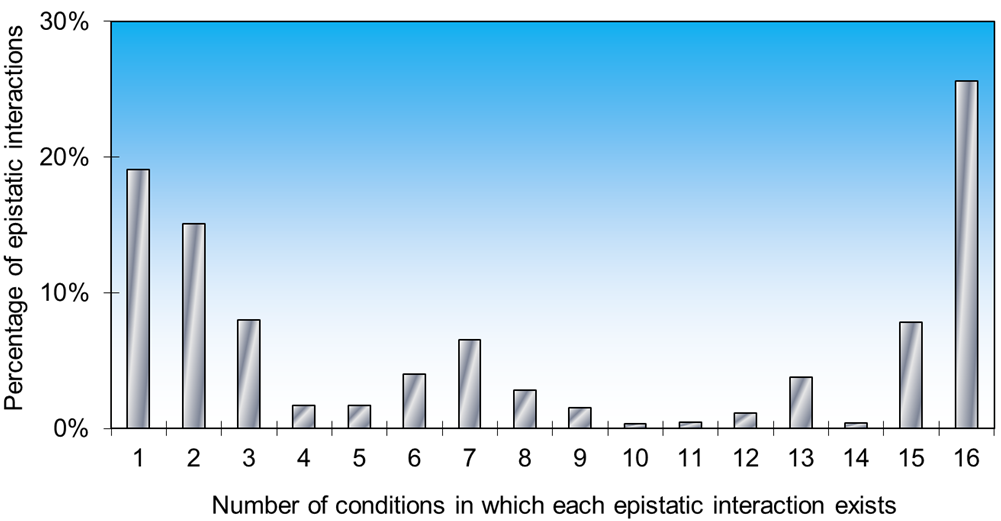

Supplement: S4 Fig — Distribution for the number of conditions in which each epistatic interaction exists. Note that about 26% of epistatic relations are extremely stable (the very right bar) and about 19% are extremely dynamic (the very left bar). (TIF) [file pone.0114911.s004.tif]
